# Correlating work hardening with co-activation of stacking fault strengthening and transformation in a high entropy alloy using in-situ neutron diffraction

**DOI:** 10.1038/s41598-020-79492-8

**Published:** 2020-12-17

**Authors:** M. Frank, S. S. Nene, Y. Chen, B. Gwalani, E. J. Kautz, A. Devaraj, K. An, R. S. Mishra

**Affiliations:** 1 Department of Materials Science and Engineering, University of North Texas, Denton, TX 76207 USA; 2 Department of Metallurgical and Materials Engineering, Indian Institute of Technology, Jodhpur, 342037 India; 3 Neutron Scattering Division, Oak Ridge National Laboratory, Oak Ridge, TN 37830 USA; 4 Physical and Computational Sciences Directorate, Pacific Northwest National Laboratory, Richland, WA 99352 USA; 5 National Security Directorate, Pacific Northwest National Laboratory, Richland, WA 99352 USA

**Keywords:** Mechanical properties, Metals and alloys

## Abstract

Transformation induced plasticity (TRIP) leads to enhancements in ductility in low stacking fault energy (SFE) alloys, however to achieve an unconventional increase in strength simultaneously, there must be barriers to dislocation motion. While stacking faults (SFs) contribute to strengthening by impeding dislocation motion, the contribution of SF strengthening to work hardening during deformation is not well understood; as compared to dislocation slip, twinning induced plasticity (TWIP) and TRIP. Thus, we used in-situ neutron diffraction to correlate SF strengthening to work hardening behavior in a low SFE Fe_40_Mn_20_Cr_15_Co_20_Si_5_ (at%) high entropy alloy, SFE ~ 6.31 mJ m^−2^. Cooperative activation of multiple mechanisms was indicated by increases in SF strengthening and γ-f.c.c. → ε-h.c.p. transformation leading to a simultaneous increase in strength and ductility. The present study demonstrates the application of in-situ, neutron or X-ray, diffraction techniques to correlating SF strengthening to work hardening.

## Introduction

Stacking faults (SFs) formed in metals and alloys are important to their deformation behavior and mechanical properties^1–3^. In face-centered cubic (FCC) metals, SFs are interruptions in the perfect (ABCABC) sequential layering of the (111) crystallographic planes, formed by either dissociation of a perfect dislocation into two partial dislocations, or by emission of partial dislocations from grain boundaries^4^. Typically the width of SFs are measured using weak beam-dark field diffraction in the transmission electron microscope (TEM)^5–8^, however SFs have also been resolved using electron contrast channel imaging (ECCI) in the scanning electron microscope (SEM)^9–12^.

Beside the SF width, other characteristic structural dimensions such as the interspacing between two SFs, i.e. SF interspacing (L_SF_), have been measured using X-ray diffraction (XRD)^13^. Importantly, the spacing between SFs has been correlated with increases in strength enhancement in various alloy systems. Stacking fault strengthening (σ_SF_) was studied in hexagonal close-packed (HCP) Mg^14,15^ alloys, FCC Cu-Al alloys^16^ and FCC Co alloys^13^ using TEM and XRD. These studies showed that the decrease in L_SF_ corresponded to an increase in yield strength due to SF-dislocation interactions. Closely spaced SF arrays contribute to strengthening by acting as barriers to dislocation motion, similar to other stable boundaries. For example, Galindo-Nava et al.^17^ suggested that deformation would also result in a decrease in twin interspacing, which was supported by ex-situ measurements using TEM. However, stacking fault strengthening has received relatively less attention as compared to slip, twinning-(TWIP) and transformation-induced plasticity (TRIP). While changes in SF dimensions have been predicted^18^, to the best of the authors’ knowledge, direct experimental evidence of deformation-induced decreases in SF interspacing, and thus SF strengthening, by an in-situ observation has not been reported. Consequently, there is a gap in the understanding of how SFs contribute to enhanced work hardenability during deformation. While TRIP lead to improvement in ductility, the simultaneous strengthening would require alternate approaches to impeding dislocation motion^19^. Thus, understanding how the activation of SF strengthening alongside TRIP contributes to overcoming the conventional strength-ductility paradigm becomes pertinent to design of advanced structural alloys.

This understanding gap likely stems from challenges in measuring SF dimensions, i.e. width or interspacing, in a specimen of a size that work hardening behavior can be simultaneously measured and correlated with deformation mechanisms. These two specimen types differ in size by orders of magnitude. For instance, SFs typically are characterized using the transmission electron microscope which requires electron transparency to avoid attenuation, less than 100 nm thick^20^. On the other hand, samples greater than 1 mm thick are often used in research to correlate deformation mechanisms to work hardening response due to the effects of grain size to thickness ratio on hardening behavior^21,22^. This difference can be overcome in in-situ experiments which use high energy X-rays or neutron. Nonetheless, the number of studies which correlate the SF strengthening deformation mechanism to work hardening behavior using in-situ approaches of this type are limited.

Recently a number of studies have investigated low SFE high entropy alloys using in-situ neutron diffraction, demonstrating the ability to relate macroscopic stress–strain response with multiple deformation mechanisms^5,23–28^. Thus, to gain a better understanding of the deformation behavior in both phases, the strengthening in the γ-f.c.c. phase by SFs was evaluated using in situ neutron diffraction in the present study. Contrary to thinner specimens typically required to study SFs, bulk tensile specimens (~ 135 mm^3^ gauge volume) of the metastable Fe_40_Mn_20_Cr_15_Co_20_Si_5_ (in at%) HEA specimen were deformed on the VULCAN neutron diffractometer^29^ at the Spallation Neutron Source (SNS) at Oak Ridge National Laboratory. The activation of SF strengthening and transformation induced plasticity (TRIP) clearly provided real-time evidence of their cooperative behavior and contributions to simultaneous enhancement of strength and ductility. While the relationship between SF strengthening and SF interspacing was previously established^13–15^, we use in-situ neutron diffraction to capture this phenomenon in real time for the first time and demonstrate the combined importance of activating multiple deformation mechanisms to overcoming the strength-ductility tradeoff.

## Results and discussion

**Figure 1:**
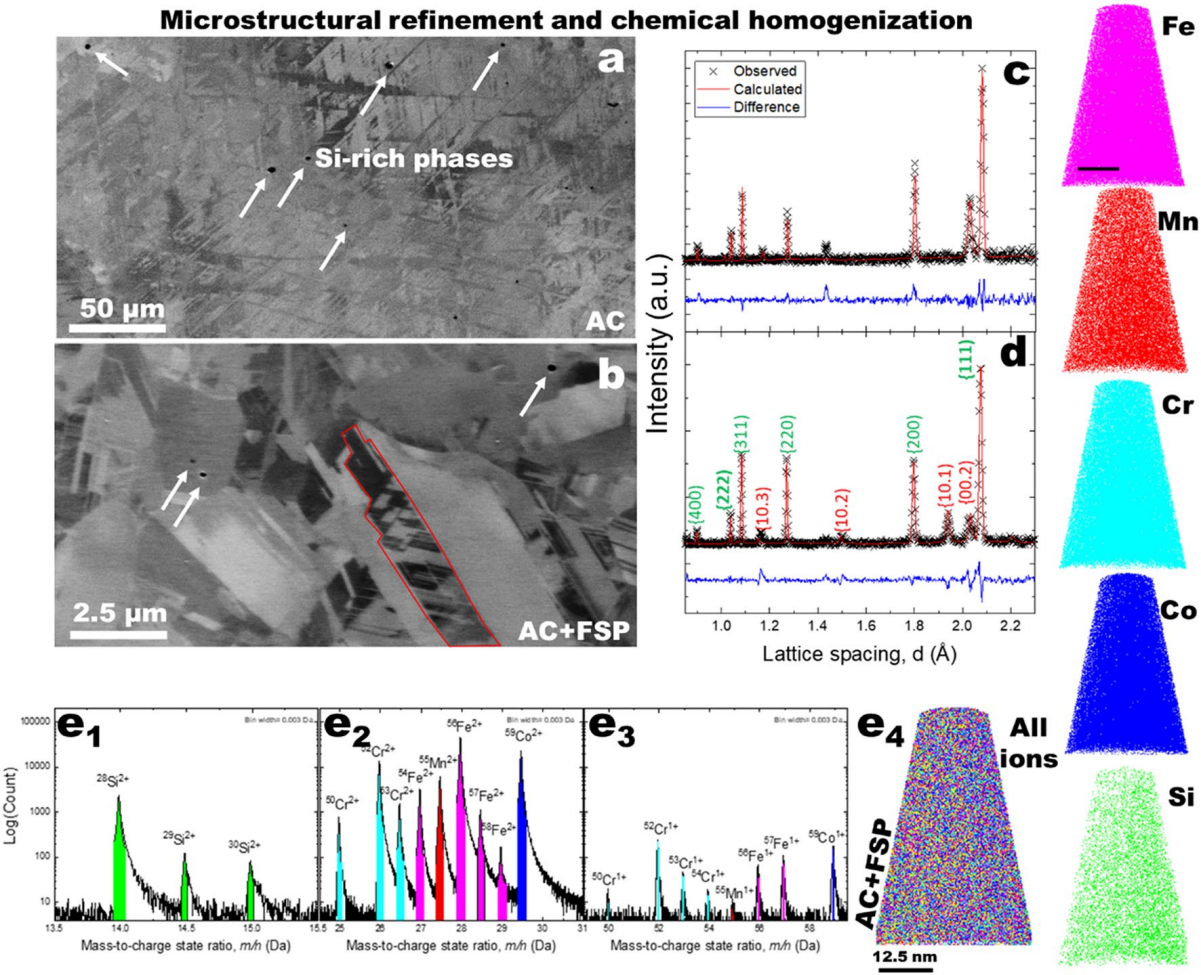
Evaluation of microstructural evolution and chemical homogenization of Fe_40_Mn_20_Cr_15_Co_20_Si_5_ alloy before tensile deformation. Secondary electron SEM images of (**a**) as-cast (AC), and (**b**) grain-refine friction stir processed (AC + FSP) conditions demonstrating the microstructure refinement following friction stir processing. Further neutron diffraction traces from the two conditions, (**c**) AC, and (**d**) AC + FSP, conclusively showed the presence of γ-f.c.c. and ε-h.c.p. phases in both the conditions. Peaks labeled green and red for γ-f.c.c. and ε- h.c.p. phases, respectively. Atom probe tomography mass spectra with prominent peaks labeled, including: (**e**_**1**_) Si ions, transition metal ions in the 2 + charge state and (**e**_**3**_) transition metal ions in the 1 + charge state. Scale bars in (**a**), (**b**) and (**e**_**4**_) are 50 μm, 2.5 μm and 12 nm, respectively.

The microstructures for both conditions prior to deformation are presented in Fig. 1a,b. The AC condition exhibited coarse grains larger than 100 µm, with martensitic laths observed throughout the microstructure, while the AC + FSP condition was significantly grain refined. Following casting and FSP, both FCC and HCP peaks were identified using neutron diffraction, Fig. 1c,d and Fig. S1 in the supplementary document for direct grain size comparison to AC. However, micrographs revealed the formation of Si-rich particles following casting, Fig. 1a. With the formation of these Si particles, depletion of Si in the matrix is likely, the stacking fault energy of the matrix would be expected to increase^23^. To investigate the Si content, energy dispersive X-ray spectroscopy (EDS) maps for AC and AC + FSP are shown in the supplementary document, Figs. S2 and S3, respectively. The measured Si content was observed to be lower than the nominal value in the AC condition, however upon FSP the Si content in the matrix increased suggesting dissolution of the Si-rich phase occurred during FSP. To evaluate the content further, atom probe tomography (APT) was used to further evaluate the spatial distribution of elements in the AC + FSP condition. Uniquely, APT has the ability to measure and visualize the composition and distribution of elements in three dimensions. The mass spectra and ion map are shown in Fig. 1e_1_–e_3_ and Fig. 1e_4_, respectively. Ions of each of the constituent elements in the alloy were captured and are represented in their cumulative total in the plots. Considering the aim to determine the Si content, it is important to mention that lower fractions of Si (73%) and Mn (76%) relative to the observation by APT (details in the Table S1 in the supplementary document). It is expected that the measured fractions are due to the effects of field ion evaporation, and that the actual fractions of each are similar to EDS results. Discrepancies in measured content by APT have been reported for Si^30^ and Mn^31^ ions in both steels and HEAs and have been discussed in further detail in the supplementary document, Fig. S4.

Aside from the measured alloying content, significant microstructural refinement was observed in the AC + FSP condition, resulting in both a reduction in the size of the Si-rich particles and an average grain size of 6 µm. The refinement and dissolution observed was owing to severe plastic deformation during FSP, which also led to a change in texture and phase proportions captured by neutron diffraction. Prior to deformation, diffraction patterns for each condition revealed dual phase γ-f.c.c. and ε-h.c.p. microstructures in Fig. 1c,d, denoted by {hkl} and {hk.l}, respectively. Comparing AC to AC + FSP, significant changes in peak intensity were observed following FSP. For example, the changes in intensity of the ε-h.c.p. {10.1} and {00.2} were observed, where the intensity of the {00.2} peak decreased significantly after FSP. The observed decrease demonstrated the effect of high strain and strain rates during FSP on the volume of grain orientations captured by neutron diffraction^23,32^. Additionally, the {10.1} peak was not observed in the AC condition whereas it was clearly captured after FSP as a result of severe plastic deformation at high temperatures. In line with this, the intensity of the γ-f.c.c. peaks changed indicated by the variation in peak when comparing both specimens. Specifically, the comparison of normalized peak intensities, by comparing the I_220_/I_111_ in both conditions, showed that along with grain refinement, FSP increased the fraction of {220} grains captured. In addition, we were also able to resolve the higher multiplicity peaks γ-f.c.c. peaks, such as {222} and {111} pair, to measure the lattice spacings, d_222_ and d_111,_ respectively. While lattice spacing is typically used to determine lattice strain during in-situ experiments, the lattice spacing can be visualized in the unit cell to understand the faulting behavior in the present discussion.

**Figure 2:**
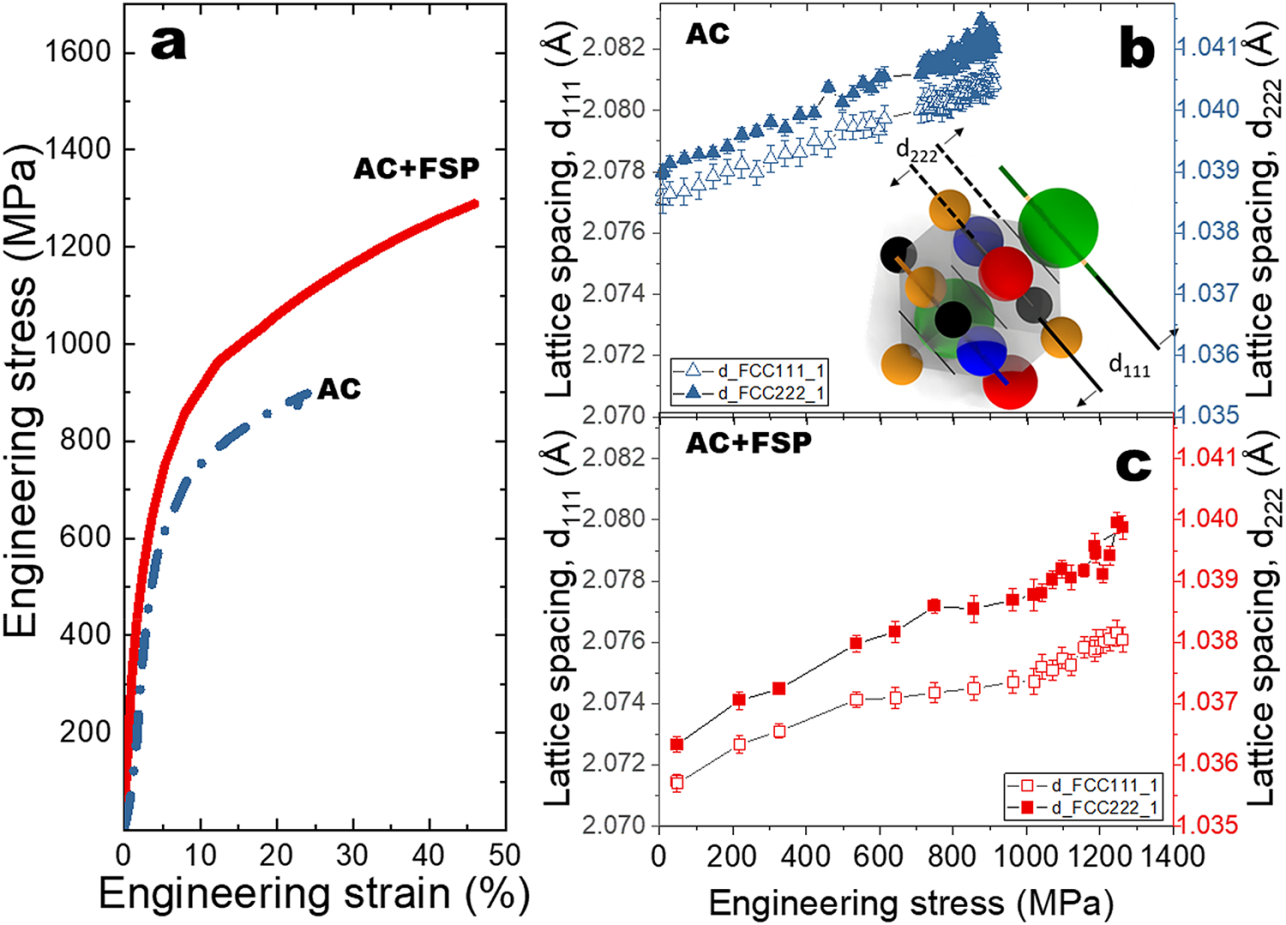
(**a**) Engineering stress–strain curves for as-cast and friction stir processed (AC and AC + FSP, respectively) conditions during in-situ neutron diffraction experiments. The lattice strain as a function of engineering stress for: (**b**) AC, and (**c**) AC + FSP conditions. Here lattice spacings are shown to correlate with the schematic representation of d_222_ and d_111_ planes and Eq. 1 with Figures (**b**) and (**c**).

The tensile stress–strain responses of the AC and AC + FSP conditions were captured during in-situ neutron diffraction experiments. Comparing the two tensile curves (Fig. 2a), FSP led to a substantial increase in strength and ductility. In previous studies on metastable HEAs exhibited dynamic stress partitioning between phases leading to activation of ε-h.c.p. related mechanisms and delayed activation of TRIP^23,33^. As we hypothesized that SFs contribute prior to the onset of transformation, we probed the changes in lattice spacing to determine the contribution of σ_SF_.

The deformation-induced increases in lattice spacings were investigated as a function of applied stress to evaluate the contribution of SFs to overall strengthening. Thus, the anisotropic deformation response between d_222_ and d_111_ in the low SFE γ-f.c.c. phase suggested faulting was captured in real-time. Faulting was observed by the differences in the evolution of lattice strain for d_222_ and d_111_ in each plot, understood by Eq. 1, which are considered to be crystallographically equivalent^34^. In the absence of anisotropy, the lattice spacing of the {222} is expected to be 50% of the {111} for the γ-f.c.c. unit cell, (inset of Fig. 2b) based on Eq. 1:

1 $$d^{2} = { }\frac{{a^{2} }}{{h^{2} + k^{2} + l^{2} }}$$

where *d* and *a* are the lattice spacing and lattice constant, whereas together h, k, and l denote the set of crystallographic planes. For instance, taking a γ-f.c.c. lattice constant of 3.60 Å the calculated *d* for the {111} and {222} planes are determined to be 2.078 Å and 1.039 Å, respectively.

In Fig. 2b,c, the ordinate axis for the {222} (right axis) is 50% of that for {111} (left axis). Considering that the axis is scaled to half, the lattice spacing as a function of applied stress would be expected to overlap based on Eq. 1, and the lack of overlap would indicate faulting. The observed anisotropy confirmed the occurrence of faulting. Furthermore, comparing the AC and AC + FSP conditions it is apparent that the initial lattice spacings were different for different processing conditions. It is suspected that this is a result of the different processing paths, as strain and temperatures in FSP are significantly different from those experienced during casting and quenching. This change in structure resulting from the combined effects of strain and temperature have also been observed in other low SFE Fe-based shape memory alloys in previous studies^35^. It is known that both strain and temperature will affect the structure. Recent studies demonstrated that processing or tensile deformation can lead to changes in both lattice constants *a* and peak broadening^23,36^.

**Figure 3:**
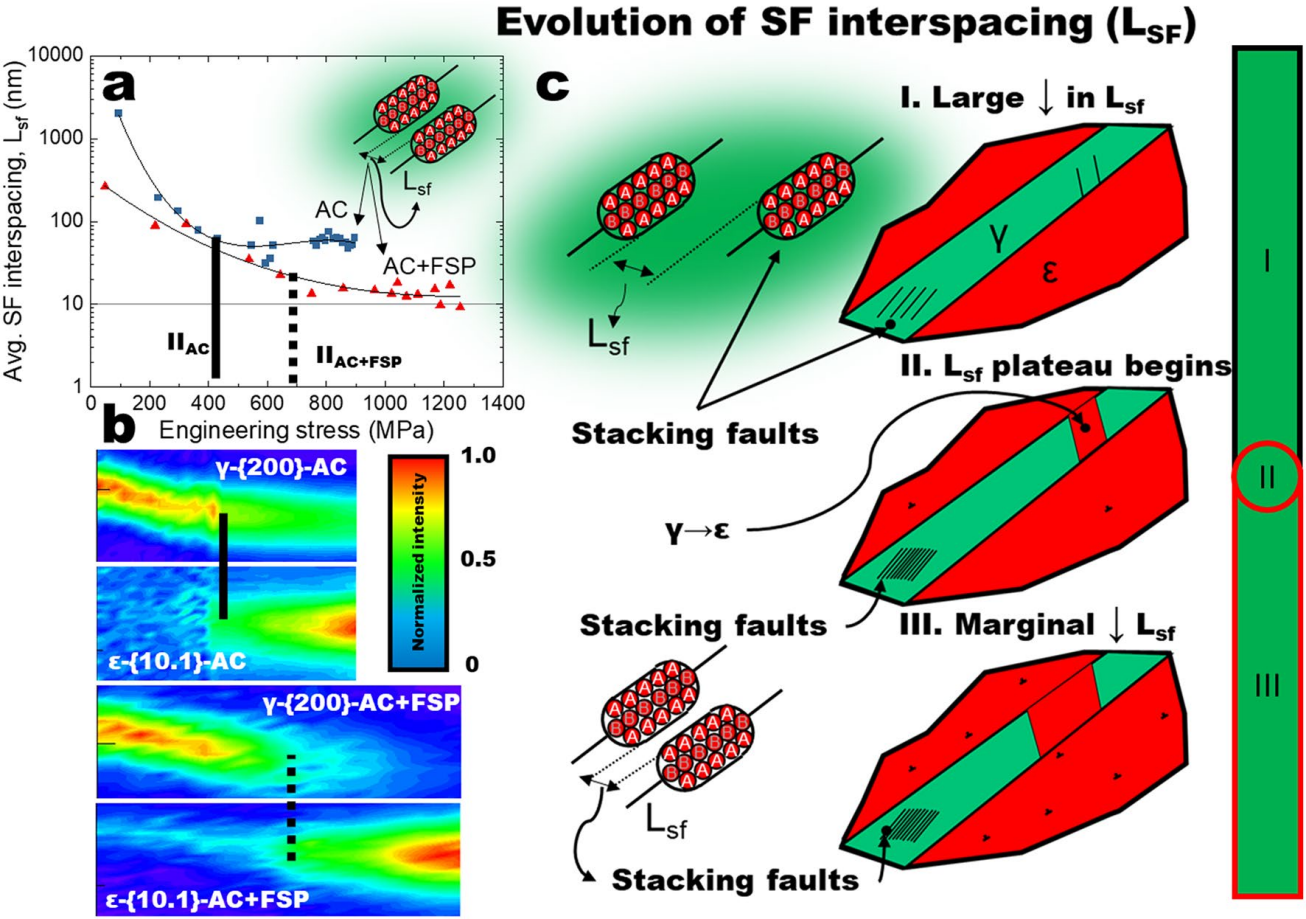
Mechanism of cooperative SF strengthening and transformation. (**a**) In-situ measurement of stacking fault interspacing, L_sf_, as a function of engineering stress. (**b**) Intensity changes indicating transformation in the as-cast (AC) and friction stir processed (AC + FSP) conditions by decrease in the γ-f.c.c. {200} intensity and an increase in the ε-h.c.p. {10.1}. (**c**) Schematic representation of SF and transformation behavior during tensile deformation. In stage I the initial SF interspacings are relatively large, L_sf_ > 100 nm for both conditions. As tensile deformation continues to stage II (initiation marked by solid (AC) or dotted (AC + FSP) lines the decrease in L_sf_ slows indicated by the change in slope between stages I and II. Subsequently, stage III is characterized by a relatively constant value of L_sf_.

Consistent with the expectation based on previous studies^23,36^, during tensile deformation, both conditions exhibited increases in lattice spacings. Importantly, the differences in the evolution of lattice spacing support the idea that the L_SF_ would behave differently in response to deformation. Prior to deformation, we also observed a large difference in L_SF_ by comparing AC and AC + FSP conditions (Fig. 3a). The difference in initial L_SF_ likely was related to severe plastic deformation imposed during FSP, leading to the formation of a high density of faults. During tensile deformation, initially the decrease in L_SF_ as a function of applied stress was relatively intense (Fig. 3a). The decrease in L_SF_ slowed, marked by a change in slope approaching plastic deformation. The AC + FSP condition also exhibited a similar decreasing response, although a more delayed decrease was observed. Interestingly, at the later stages of plastic deformation, the change in SF interspacing reaches saturation, around ~ 10 nm. This interspacing was similar to that observed in the case of both Mg alloys and Co alloys previously discussed, ~ 16 nm^14^ and ~ 10 nm^13^, respectively. The implications of these results confirm that SF spacing decreases not only during elastic deformation but also could contribute to strengthening during plastic deformation.

As plastic deformation continued, the evolution of L_SF_ was investigated. With increasing applied stress, both conditions reached the lowest L_SF_ observed (Fig. 3a). This was clearly the case for AC, which exhibited a decrease to ~ 50 nm; while the AC + FSP condition decreased to ~ 10 nm. The plateau of SF interspacing was expected to be related to the onset of transformation or, more specifically, arrays of faults accumulated with spacing and critical dimensions sufficient to have been detected as HCP martensite during neutron diffraction. To further correlate the decrease in SF interspacing with the onset of transformation, we evaluated the intensity changes related to transformation, as a function of applied stress. Figure 3b shows the changes in intensity for the γ-f.c.c. {200} and ε-h.c.p. {10.1} orientations; i.e., the orientation that provides the first indication of strain-induced transformation in metastable HEAs. Strain-induced transformation was observed by the decrease in {200} intensity and the subsequent increase in {10.1} intensity. Alongside the gradual decrease in {200} intensity, a plateau in the decrease in SF interspacing coincided with the increase of the intensity of {10.1} (Fig. 3a,b). Interestingly, the plateau occurred at different L_SF_ for both AC and AC + FSP specimens. This observation suggests that the average value of SF interspacing did not govern the formation of HCP martensite, but instead independently decreased until the onset of transformation. For the strain-induced γ-f.c.c. → ε-h.c.p. transformation, SFs are well-known to be precursors to the formation of ε-h.c.p. martensite; i.e., a critical number of faults align to form arrays with a critical size and interspacing to be detected as ε-h.c.p.^17^. The onset of transformation or twinning have been associated with relieving excess stress concentrations due to dislocation pile ups^17^. The stress relief with activation of transformation can explain the plateau in SF interspacing observed in the present study. As shown schematically in Fig. 3c, faults are detected and observed to decrease in the γ-f.c.c. phase of the alloy. The three stages describe the behavior of L_SF_. In stage I, L_SF_ exhibited a steady decrease with increasing applied stress, while the transformation behavior at each instance can be observed in Fig. 3b. At stage II, or point II, the plateau of L_SF_ begins which is indicated by the circle. In stage III, the decrease in L_SF_ is relatively lower although it occurs alongside the γ-f.c.c. → ε-h.c.p. transformation. As deformation occurred, the density of faults increased with increasing applied stress. Considering two faults were present initially, understandably, the nucleation of a new fault between the two existing faults would have resulted in the observed decrease in SF interspacing. The changes in SF interspacing during plastic deformation are evaluated to understand the contribution of SFs to work hardening behavior.

**Figure 4:**
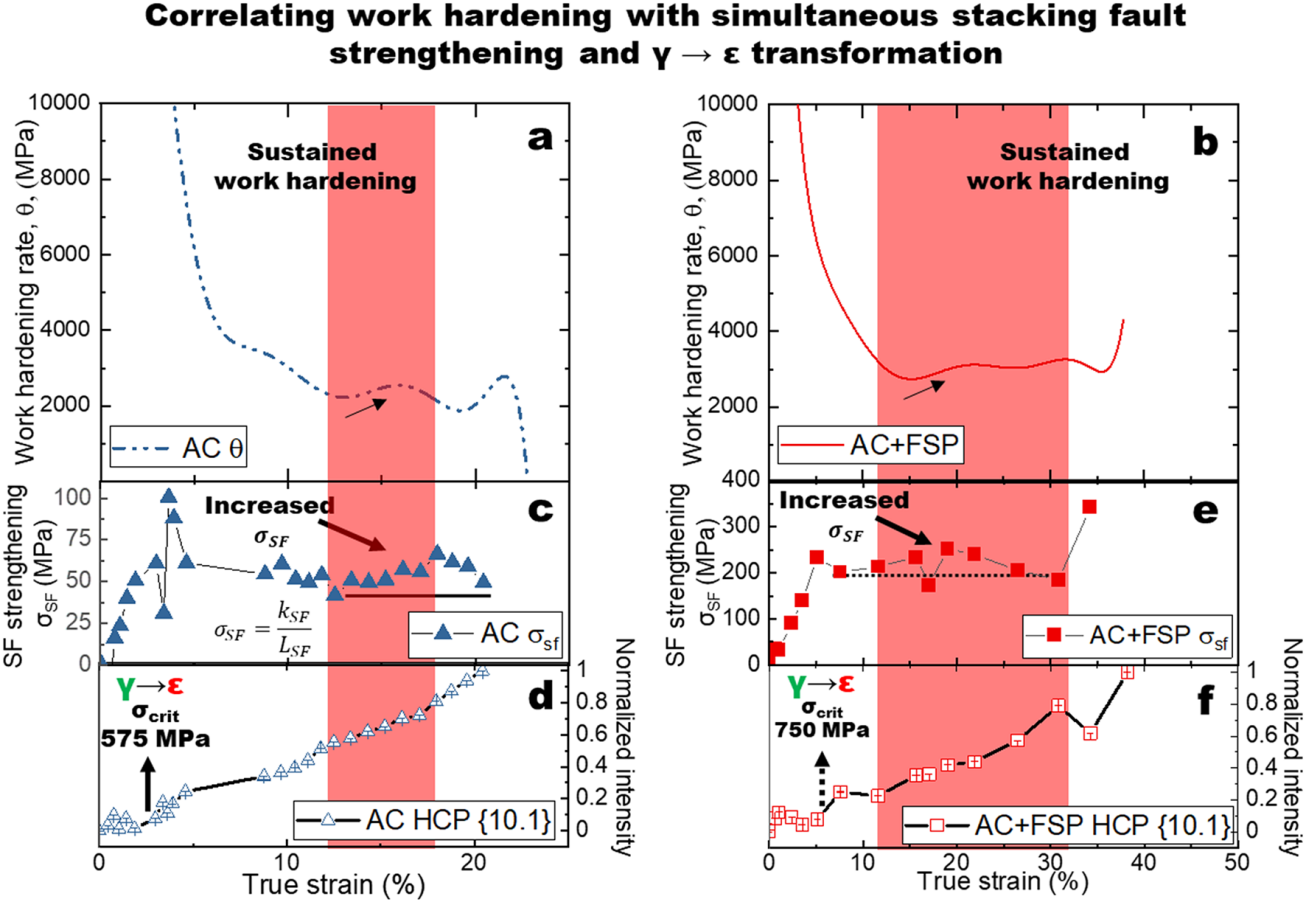
The work hardening behavior of Fe_40_Mn_20_Cr_15_Co_20_Si_5_ high entropy alloy. Work hardening curves for (**a**) as-cast (AC) and (**b**) friction stir processed (AC + FSP) conditions. The stacking fault strengthening, σ_SF_, (for (**c**) AC and (**e**) AC + FSP, respectively) and intensity of the ε-h.c.p. {10.1} intenstiy (for (**d**) AC and (**f**) AC + FSP, respectively) as a function of true strain. The transition in deformation behavior is observed by the plateau in *σ*_*SF*_ (marked by horizontal black lines) the coincident increase in the ε-h.c.p. phase fraction marked by the increase in intensity of the {10.1}.

To understand the origins of the observed increases in strength and ductility, Fig. 4 correlates the work hardening response to both SF strengthening and transformation. Figures 4a,b show the work hardening behavior of the metastable Fe_40_Mn_20_Cr_15_Co_20_Si_5_. While multiple mechanisms are activated during deformation^23^, we investigated the co-activation of SF strengthening and transformation to correlate observed enhancements of work hardening. Sustained work hardening rate (θ) of around ~ 3000 MPa was observed in AC + FSP, whereas AC exhibited a more rapid decrease. The red regions highlight the sustained σ_SF_ and increasing transformation correlated with changes in θ. Additional details for determining k_SF_, in order to determine σ_SF_, are given in the supplementary document Fig. S6. Interestingly, the σ_SF_ and the γ-f.c.c. → ε-h.c.p. transformation were observed to be related to the work hardening behavior, comparing Figs. 4a,f. The σ_SF_ for both conditions are presented in Fig. 4c,e. During the initial stages of deformation, the contribution of SFs increased, up to ~ 50 and ~ 250 MPa for AC and AC + FSP, respectively. The strengthening contribution measured was similar to FCC Co-based alloy, up to ~ 500 MPa for yield strength of about 1200 MPa^13^. It is important to mention that in previous studies of SF strengthening in HCP Mg and FCC Co alloys, no TRIP was present, and the increase of strength corresponded to a decrease in ductility. In the present study, the AC condition exhibited SF strengthening and a relatively lower fraction of transformation, Fig. S5, which can explain the lower ductility. The cooperative activation of deformation mechanisms in the present study was evidenced by increases in σ_SF_ and the subsequent onset of transformation. The σ_SF_ initially increased while the {10.1} intensity (the γ-f.c.c. → ε-h.c.p. transformation) exhibited no change, Fig. 4c,e.

**Figure 5:**
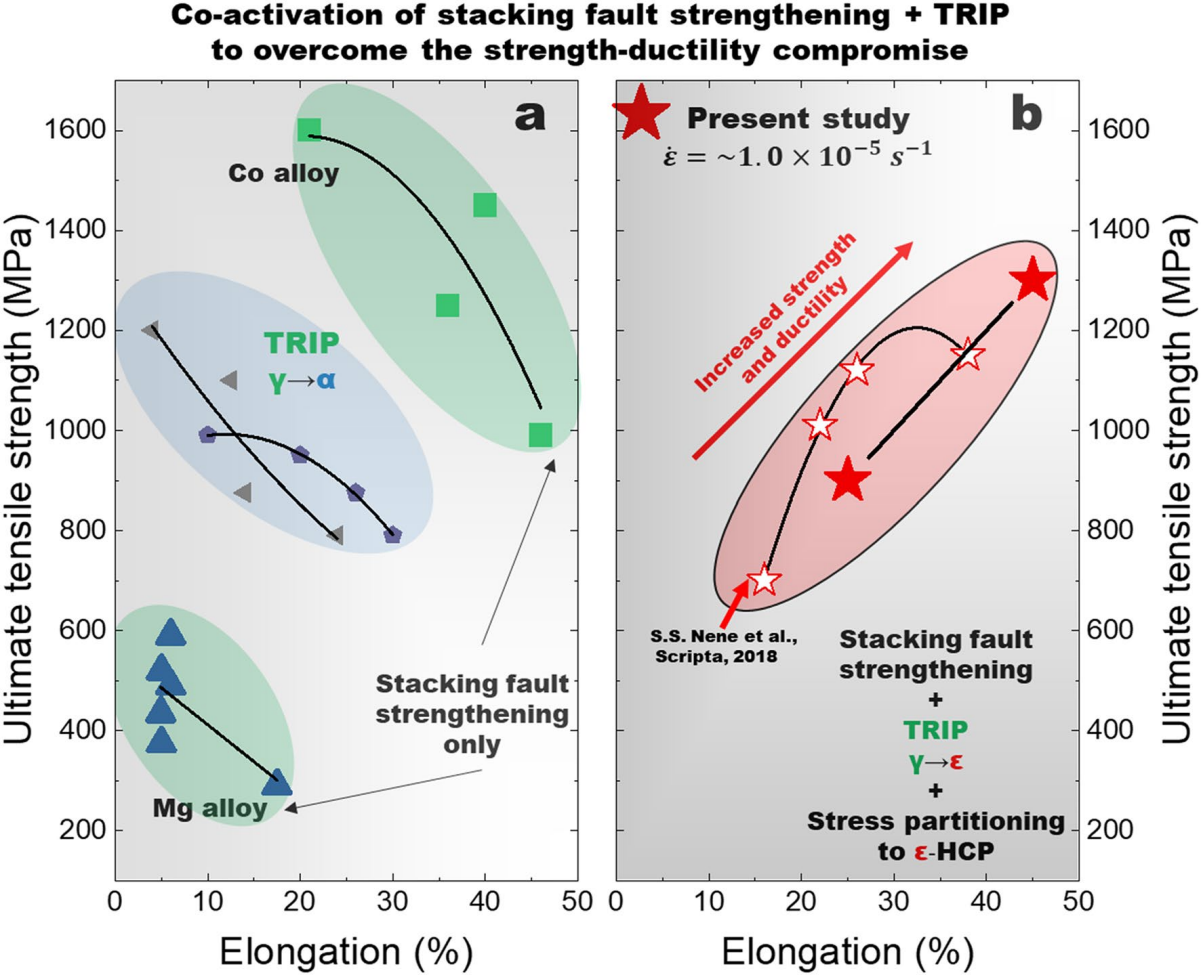
The combined effect of stacking fault strengthening and transformation induced plasticty (TRIP) to overcome the strength-ductility tradeoff. Strength-ductility comparison shows that activation of both SF strengthening and TRIP lead to the simultaneous increase in strength and ductiltiy observed in the present Fe_40_Mn_20_Cr_15_Co_20_Si_5_ high entropy alloy. (**a**) Comparison of strength ductility combinations of TRIP steels^37,38^ exhibiting the strain induced austenite to α martensite phase transformation with both stacking fault strengthened Mg^14^ and Co^13^ alloys ($\dot{\varepsilon } \approx 1 \times 10^{ - 3} s^{ - 1}$). (**b**) Comparison of the Fe_40_Mn_20_Cr_15_Co_20_Si_5_ high entropy alloy in the present study ($\dot{\varepsilon } \approx 1 \times 10^{ - 5} s^{ - 1}$) with the literature^32^.

During plastic deformation, both mechanisms are active, indicated by the sustained σ_SF_ and continuously increasing {10.1} intensity result in overcoming the strength-ductility compromise. The cooperative behavior observed during plastic deformation was likely related to complex stress partitioning and stress relief on SFs enabled by the onset of TRIP^17^. The enhanced work hardenability can be explained by the cooperation of multiple deformation mechanisms during straining^23,33^. SF strengthening was sustained and the {10.1} intensity was observed to increase in unison. The sustained contribution of SFs originates from the ability to impede dislocation with fine interspacing. Regions highlighted in red indicate sustained/increasing work hardening rate was observed. To further demonstrate the implications of co-activation of the two mechanisms, Fig. 5 compares the strength-ductility combinations of alloys which undergo either SF strengthening or TRIP, with the present Fe_40_Mn_20_Cr_15_Co_20_Si_5_ HEA where both deformation modes were observed to occur simultaneously. The Mg and Co alloys undergo stacking fault strengthening in the absence of TRIP which resulted in a decrease in ductility with the increase in strength. Similarly, a decrease in ductility was observed for TRIP steels that exhibited the strain-induced austenite to α martensite phase transformation. However, it is important to note that SFs would have also formed in the TRIP steels. In the present study, we observed that less transformation corresponded to less ductility (by comparison of fractured AC and AC + FSP conditions). Based on these results, it would be expected that SFs would have contributed to strengthening in TRIP steels, while transformed fraction would have been decreasing with the increase in strength. This question requires additional research to draw any conclusions regarding comparisons of TRIP HEAs with TRIP steels. Nonetheless, the observations from previous studies and the comparison of ductility in the AC and AC + FSP conditions suggest that the contributions of both SF strengthening and TRIP are important to enabling the synergistic increase in strength and ductility in metastable HEAs.

## Conclusions

In-situ neutron diffraction was used to quantify stacking fault strengthening and reveal the onset of the γ-f.c.c. → ε-h.c.p. transformation in a metastable Fe_40_Mn_20_Cr_15_Co_20_Si_5_ high entropy alloy. First, the present study demonstrates the application of in situ diffraction techniques for real-time quantification of SF strengthening in low stacking fault energy metals and alloys. Our results show that in-situ neutron diffraction can be used to measure the interspacing between SFs, L_SF_, during deformation in real-time. The L_SF_ decreased with increasing tensile deformation, to ~ 50 nm and ~ 10 nm in cast (AC) and friction stir processed (AC + FSP) conditions, respectively. SF strengthening contributions of 50 MPa and 250 MPa were determined for AC and AC + FSP conditions, respectively. The changes in L_SF_ and σ_SF_ indicate that stacking faults also contribute to strengthening during plastic deformation alongside other mechanisms. Further, the results reveal that the decay in SF interspacing slows with the onset of γ-f.c.c. → ε-h.c.p. transformation. Along with recent studies highlighting the importance of stress partitioning, a relatively low transformation was observed to correspond with a lack of ductility in the AC condition. The present results demonstrate that in addition to the contribution of grain size, initial ε-h.c.p. phase fraction, the presence of precipitate phases and mechanical twins, the γ-f.c.c. → ε-h.c.p. phase transformation being accompanied by the stacking fault strengthening deformation mode is also important to attaining simultaneous increases in both strength and ductility.

## Methods

The Fe_40_Mn_20_Cr_15_Co_20_Si_5_ alloy was cast in a vacuum induction furnace with pure metals to achieve nominal compositions, henceforth the as-cast condition (AC). The cast plate 300 × 100 × 6 mm was subjected to friction stir processing (FSP) for microstructural refinement, henceforth denoted as AC + FSP. A tungsten-rhenium tool used for processing with a shoulder diameter of 12 mm, and pin geometry consisted of root diameter, distal tip diameter, and length of the tool of 7.5 mm, 6 mm, and 3.5 mm, respectively. Tool rotation rate was 350 rotations per minute (RPM), and traverse speed was 50.8 mm/min. To characterize the extent of microstructural refinement following processing, optical microscopy was carried out on both conditions. For secondary electron (SE) images a FEI Quanta 3D FEG microscope (FEI Company, Hillsboro, Oregon) was operated at 20 kV to investigate the microstructures of both AC and AC + FSP conditions. The Quanta microscope used for imaging was a dual beam scanning electron microscope/focused ion beam (SEM/FIB) and was also used for atom probe tomography (APT) needle preparation using the FIB lift-out method^39,40^. APT was carried out using the CAMECA LEAP 4000 XHR equipped with an ultraviolet wavelength laser, 355 nm. Temperature, pulse rate, detection rate, and pulse energy were 60 K, 125 kHz, 0.003 detected ions/pulse detection rate and 60 pJ, respectively. The analysis chamber pressure was kept at ~ 2 × 10^–11^ Torr. The LEAP used here has a detector efficiency of ~ 36%. Reconstruction of APT data was carried out in CAMECA’s Integrated Visualization and Analysis Software (IVAS) version 3.8.2 for determination of composition, visualization of ion maps and subsequent analysis of the spatial distribution of elements.

The use of neutron diffraction was selected for the present study due to the penetration depth of neutrons and resolution power of crystallographically equivalent high order peaks. Tensile specimens were deformed on the VULCAN neutron diffractometer^29,41^ at the Spallation Neutron Source (SNS) at the Oak Ridge National Laboratory. A detailed description of the tensile loading procedures has been provided in prior work^23,33^. Thus, data reduction after experiments was done and analysis was performed using VDRIVE software^42^. In VDRIVE, single-peak fitting was carried out, where the peak profile function is a convolution of the back-to-back exponentials with the pseudo-Voigt function. Thus, single peak fitting was used to determine both lattice spacing and integrated intensities for various family of grains.

In time-of-flight neutron diffraction, deformation induced changes in the lattice spacing, d^hkl^, of families of grains, for example {111} and {222}, in polycrystalline materials are measured. The change in lattice spacing is used to determine lattice strain in a particular family of grains ε_hkl_:

2 $${\upvarepsilon }_{{{{\rm hkl}}}} = \frac{{{\text{d}}_{{\text{i}}}^{{{\text{hkl}}}} - {\text{d}}_{0}^{{{\text{hkl}}}} }}{{{\text{d}}_{0}^{{{\text{hkl}}}} }}$$

Equation 2 can be incorporated into the lattice strain evolution of crystallographically equivalent {111} and {222} families of grains^34^. Using the changes in lattice strain for each, the stacking fault probability can be determined using the relationship between P_SF_ and lattice strain, given by,

3 $${\upvarepsilon }_{{\left\{ {222} \right\}}} - {\upvarepsilon }_{{\left\{ {111} \right\}}} = - \frac{\sqrt 3 }{{4{\uppi }}}\frac{{\mathop \sum \nolimits_{{\text{b}}} \left( { \pm {\text{L}}_{0} } \right)}}{{{\text{h}}_{0}^{2} \left( {{\text{u}} + {\text{b}}} \right)}}{\text{P}}_{{{{\rm SF}}}}$$

The term $\frac{{\mathop \sum \nolimits_{{\text{b}}} \left( { \pm {\text{L}}_{0} } \right)}}{{{\text{h}}_{0}^{2} \left( {{\text{u}} + {\text{b}}} \right)}}$ is determined by crystallographic orientations selected as detailed in^2,34^ The {111} and {222} orientation values of + 1/4 and − 1/8, respectively, as reported, give rise to the following simplified expression relating lattice strain and P_SF_,

4 $${\text{P}}_{{{{\rm SF}}}} = \frac{1}{0.0517}\left( {{\upvarepsilon }_{{\left\{ {222} \right\}}} - {\upvarepsilon }_{{\left\{ {111} \right\}}} } \right)$$

The interspacing between SFs can be determined by analyzing changes in lattice spacing. The interspacing between SFs was determined by measurement of the d_111_ and the P_SF_, given by the following relationship^13^:

5 $${\text{L}}_{{{{\rm SF}}}} = { }\frac{{{\text{d}}_{111} }}{{{\text{P}}_{{{{\rm SF}}}} }}$$

The SF strengthening, σ_SF_, is proportional 1/L_SF_^13^, thus as the SF interspacing decreases σ_SF_ increases. The strengthening coefficient, k_SF_, is determined by the slope of the 1/L_SF_ versus yield strength plot, see supplementary Fig. S5. The σ_SF_ contribution can then be determined as a function of L_SF_ in real-time by^13–15^:

6 $$\sigma_{{{{\rm SF}}}} = { }\frac{{{\text{k}}_{{{{\rm SF}}}} }}{{L_{{{{\rm SF}}}} }}$$

## Supplementary information

Supplementary Information.
