# Supplementary material for: Correlating work hardening with co-activation of stacking fault strengthening and transformation in a high entropy alloy using in-situ neutron diffraction
Source: Sci Rep. 2020 Dec 17;10:22263. doi: 10.1038/s41598-020-79492-8 (PMC7747568; doi:10.1038/s41598-020-79492-8)
Supplement: Supplementary file 1 — Supplementary Information. [file 41598_2020_79492_MOESM1_ESM.docx]

Supplementary attachment for “**Correlating work hardening with co-activation of stacking fault strengthening and transformation in a high entropy alloy using *in-situ* neutron diffraction”**

M. Frank^a^, S. S. Nene^a,b^, Y Chen^c^, B. Gwalani^d^, E. J. Kautz^e^, A. Devaraj^d^, K. An^c^_,_ R. S. Mishra^a*^

^a^ Department of Materials Science and Engineering, University of North Texas, Denton, Texas 76207 USA

^b^ Department of Metallurgical and Materials Engineering, Indian Institute of Technology, Jodhpur, 342037 India

^c^ Neutron Scattering Division, Oak Ridge National Laboratory, Oak Ridge, Tennessee 37830 USA

^d^ Physical and Computational Sciences Directorate, Pacific Northwest National Laboratory, Richland, Washington 99352 USA

^e^ National Security Directorate, Pacific Northwest National Laboratory, Richland, Washington 99352 USA

*Corresponding author: [Rajiv.Mishra@unt.edu](mailto:Rajiv.Mishra@unt.edu)

Figure S1 – Grain refinement by friction stir processing.

Figure S2 – EDS maps for as-cast Fe_40_Mn_20_Cr_15_Co_20_Si_5_.

Figure S3 – EDS maps for friction stir processed Fe_40_Mn_20_Cr_15_Co_20_Si_5_.

Figure S4 – Atom probe tomography nearest neighbor analysis for similar and dissimilar ion pairs with Mn and Si.

Figure S5 – Comparison of before and after deformation neutron diffraction patterns.

Figure S6 – Determination of the stacking fault strengthening coefficient.

Table S1 – Nominal and measured compositions for Fe_40_Mn_20_Cr_15_Co_20_Si_5_.


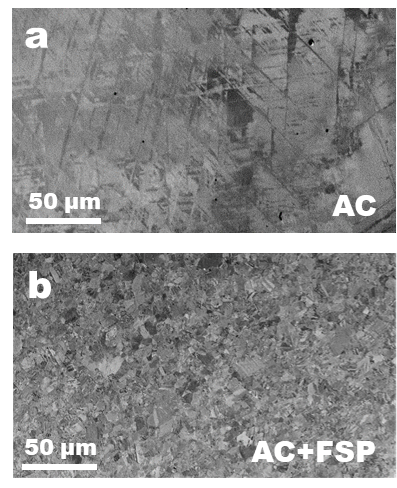


Figure S1. Grain refinement by friction stir processing (FSP). The effect of FSP on grain refinement, by comparison of cast (AC) and friction stir processed (AC+FSP) Fe_40_Mn_20_Cr_15_Co_20_Si_5_ high entropy alloy (at. %).


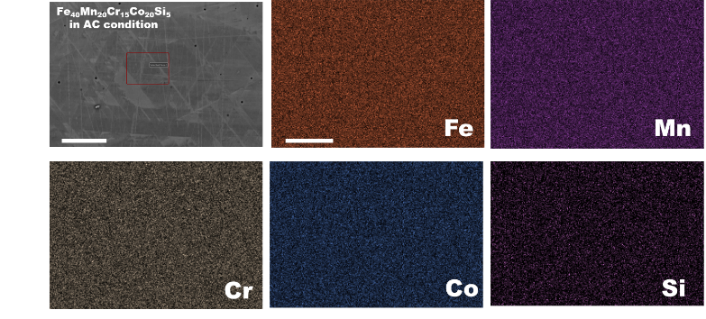


Figure S2. Secondary electron (SE) image of the as-cast (AC) Fe_40_Mn_20_Cr_15_Co_20_Si_5_. EDS maps revealing homogeneous distribution of Fe, Mn, Cr, Co and Si at SEM level. Scale bars is 10 μm.


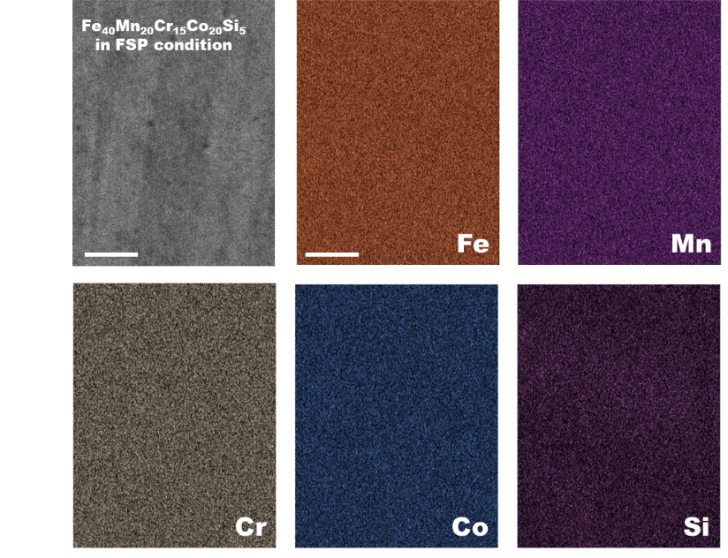


Figure S3. Secondary electron (SE) image of Fe_40_Mn_20_Cr_15_Co_20_Si_5_ follwing FSP. EDS maps revealing homogeneous distribution of Fe, Mn, Cr, Co and Si at SEM level. Scale bars is 10 μm.


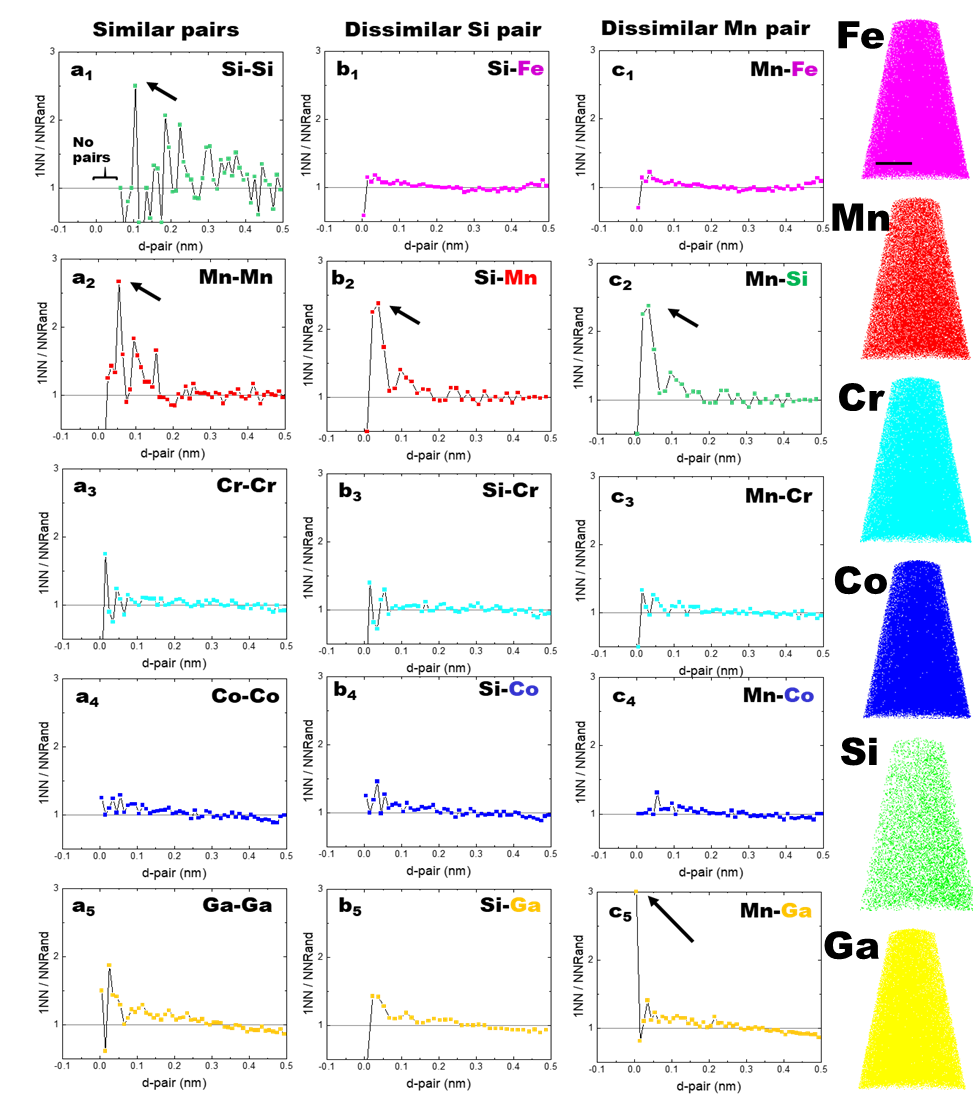


Figure S4. APT ion maps and nearest neighbor analysis showing the frequrency that two specific ions are detected a certain distance from one another. Nearest neighbor analysis suggests that short range clustering would have occurred during field ion evaporation. These observations are similar to observations made in the literature and expected to be related to discrepancies in the bulk compositon measurment from APT. (a_1_ – a_5_) similar atom and for disimilar pairs of atoms with Si (b_1_ – b_5_) and Mn (c_1_ – c_5_), respectively. Ratio of 1NN / 1NN_Rand_ greater than 1 would indicate short range clustering. The similar ion pairs Mn-Mn, Si-Si exhibit a tendency for short range cluster while dissimilar pairs of Mn-Si and Mn-Ga were determined to have the highest 1NN / 1NN_Rand_. (d) APT ion maps showing the observed distribution of all and individual elements detected.


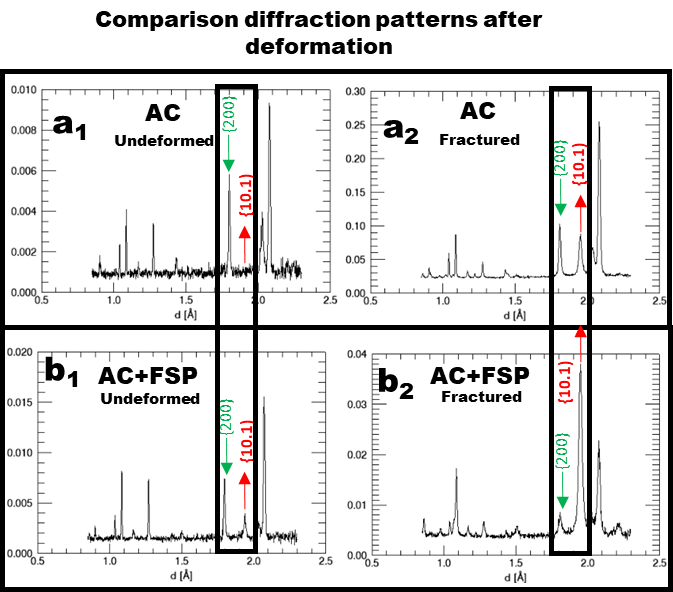


Figure S5. Neutron diffraction patterns following tensile deformation showing the relatively lower transformation observed in as-cast (AC) as compared to the friction stir processed condition (AC+FSP). AC in (a_1_) undeformed and (a_2_) fractured conditions while AC+FSP in (b_1_) undeformed and (b_2_) fractured conditions are also shown. Transformation is largely captured by the decrease in FCC {200} and increase of the HCP {10.1} peak during deformation, demonstrating relatively small transformation was observed in the case of the AC condition.

Table S1 – Nominal and measured compositions (by EDS and APT) for friction stir processed Fe_40_Mn_20_Cr_15_Co_20_Si_5_ CS-HEA.

| Fe_40_Mn_20_Cr_15_Co_20_Si_5_  (in at. %) | Fe | Mn | Cr | Co | Si | Ga* |
| --- | --- | --- | --- | --- | --- | --- |
| Nominal | $40$ | $20$ | $15$ | $20$ | $5$ | - |
| As-cast (EDS) | $40.80$ | $20.00$ | $14.80$ | $20.1$0 | $4.30$ | - |
| FSPS350 (EDS) | $38.60$ | $20.07$ | $15.95$ | $19.24$ | $6.14$ | - |
| FSPS350 (APT) | $46.65$ | $5.34$ | $13.49$ | $19.48$ | $1.20$ | $10.29$ |

* From APT sample preparation by focused ion beam (FIB) milling with Ga source.

Some previous APT studies have reported discrepancies in the measured composition of materials containing Mn and several elements found in groups III – VI. Studies have attributed these discrepancies to the effects of field ion evaporation, which have been evidenced by the short-range clustering of solutes. It has been discussed that the formation of both homogeneous and heterogeneous molecular ions occurs and are expected to detriment the accuracy of the composition measurement ^1^. In the present work, it was interesting that Si and Mn had the largest tendency to cluster, and also were the elements with the largest discrepancies. The short-range clustering was identified by nearest neighbor analysis in the present study using IVAS software for APT reconstruction. The short-range clustering observed is similar to that reported in the literature, and could explain the measured inconsistencies, supplementary Fig. S4. We observed that similar Mn-Mn and Si-Si ion pairs had a tendency to cluster, while dissimilar ion pairs of Mn-Si and Mn-Ga also showed significant clustering. Due to the prospect that the observed inconstancies are likely related to the effects of field ion evaporation, further investigation of this observation is ongoing and has been planned for a subsequent study.


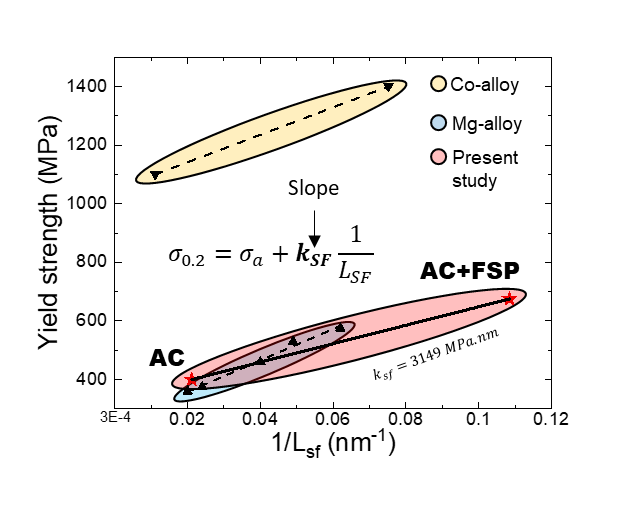


Figure S6. Determination of the stacking fault strengthening coefficient. The method used in previous studies^2–4^ on stacking fault strengthening was used to determine the stacking fault strengthening coefficient (k_sf_) by the slope similar to the Hall-Petch relationship.

References

1. Gault, B., Danoix, F., Hoummada, K., Mangelinck, D. & Leitner, H. Impact of directional walk on atom probe microanalysis. *Ultramicroscopy* (2012) doi:10.1016/j.ultramic.2011.06.005.

2. Jian, W. W. *et al.* Ultrastrong Mg alloy via nano-spaced stacking faults. *Mater. Res. Lett.* (2013) doi:10.1080/21663831.2013.765927.

3. Jian, W. W. *et al.* Physics and model of strengthening by parallel stacking faults. *Appl. Phys. Lett.* (2013) doi:10.1063/1.4822323.

4. Yamanaka, K., Mori, M., Sato, S. & Chiba, A. Stacking-fault strengthening of biomedical Co-Cr-Mo alloy via multipass thermomechanical processing. *Sci. Rep.* (2017) doi:10.1038/s41598-017-10305-1.
